# Correction: Zhang et al. Circular Nucleic Acids Act as an Oncogenic MicroRNA Sponge to Inhibit Hepatocellular Carcinoma Progression. *Biomedicines* 2025, *13*, 1171

**DOI:** 10.3390/biomedicines13102394

**Published:** 2025-09-29

**Authors:** Qianyi Zhang, Pengcheng Sun, Guang Hu, Xuanyao Yu, Wen Zhang, Xuan Feng, Lan Yu, Pengfei Zhang

**Affiliations:** 1 School of Life Sciences, Tianjin University, Tianjin 300072, China; zhangyishan0618@163.com; 2 Institutes of Biomedical Sciences, School of Life Sciences, Inner Mongolia University, Hohhot 010070, China; yuxuanyao369@163.com (X.Y.); 111984066@imu.edu.cn (W.Z.); 14747346480@163.com (X.F.); 3 Hangzhou Institute of Medicine, Chinese Academy of Sciences, Hangzhou 310018, China; hug1109@hnu.edu.cn; 4 Department of Obstetrics and Gynecology, The Fourth Affiliated Hospital of Harbin Medical University, Harbin 150001, China; hmu601784@hrbmu.edu.cn; 5 School of Biomedical Sciences, Hunan University, Changsha 410082, China; 6 Institute of Basic Medicine, Inner Mongolia Academy of Medical Sciences, Hohhot 010010, China; 7 Clinical Medical Research Center, Inner Mongolia Key Laboratory of Gene Regulation of the Metabolic Diseases, Inner Mongolia People’s Hospital, Hohhot 010010, China

## Text Correction

There was an error in the original publication [1]. Reference 25 (Yan et al.) needs to be removed, since the article has been retracted (https://pubmed.ncbi.nlm.nih.gov/36377386/ accessed on 10 August 2025). Regarding reference 25 in the original publication, its primary function in the article is to emphasize the pro-apoptotic and anti-cancer effects of ANGPTL1. Notably, this conclusion is further corroborated by references 24, 26, and 27. In addition, we have conducted further research and identified a new reference [2] to enhance the persuasiveness of our work, which will substitute the retracted one.

A correction has been made to the third sentence of the fourth paragraph in Section 3.2, together with the replacement of reference 25.

The corrected sentence has been modified as follows: “Previous studies have shown that ANGPTL1 promoted apoptosis by suppressing the STAT3/Bcl-2-mediated anti-apoptotic pathway and reduced cell migration and invasion [24–27].” The order of the cited references remains unchanged.

The authors state that the scientific conclusions are unaffected. This correction was approved by the Academic Editor. The original publication has also been updated.
