# Supplementary material for: Circular Nucleic Acids Act as an Oncogenic MicroRNA Sponge to Inhibit Hepatocellular Carcinoma Progression
Source: Biomedicines. 2025 May 11;13(5):1171. doi: 10.3390/biomedicines13051171 (PMC12109070; doi:10.3390/biomedicines13051171)
Supplement: Supplementary file 1 [file biomedicines-13-01171-s001.zip › biomedicines-3586950-supplementary.pdf]

biomedicines

MDPI

Supplementary Materials:

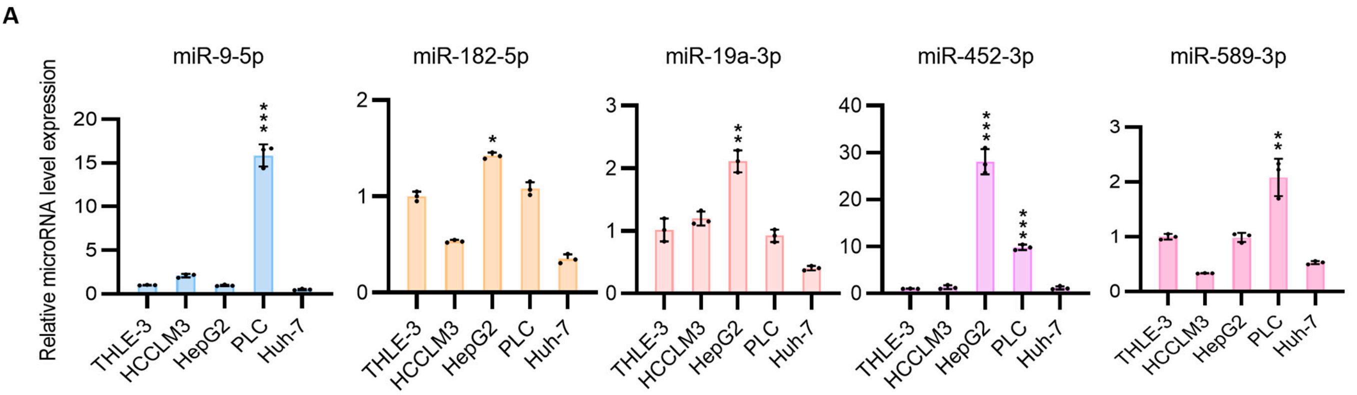

Figure S1. Relative Quantitative Expression of OncomiRs in HCC Cells and Normal Liver Cells.

(**A**) Quantitative polymerase chain reaction (qPCR) analysis of the miRNA expression of miR-9-5p, miR-182-5p, miR-19a-3p, miR-452-3p, miR-589-3p in normal cells (THLE-3) and tumor cells (HepG2, HCCLM3, PLC and Huh-7). Statistical significance was determined by Student's t-tests (n ≥ 3, **p* < 0.05, ***p* < 0.005, ****p* < 0.001).

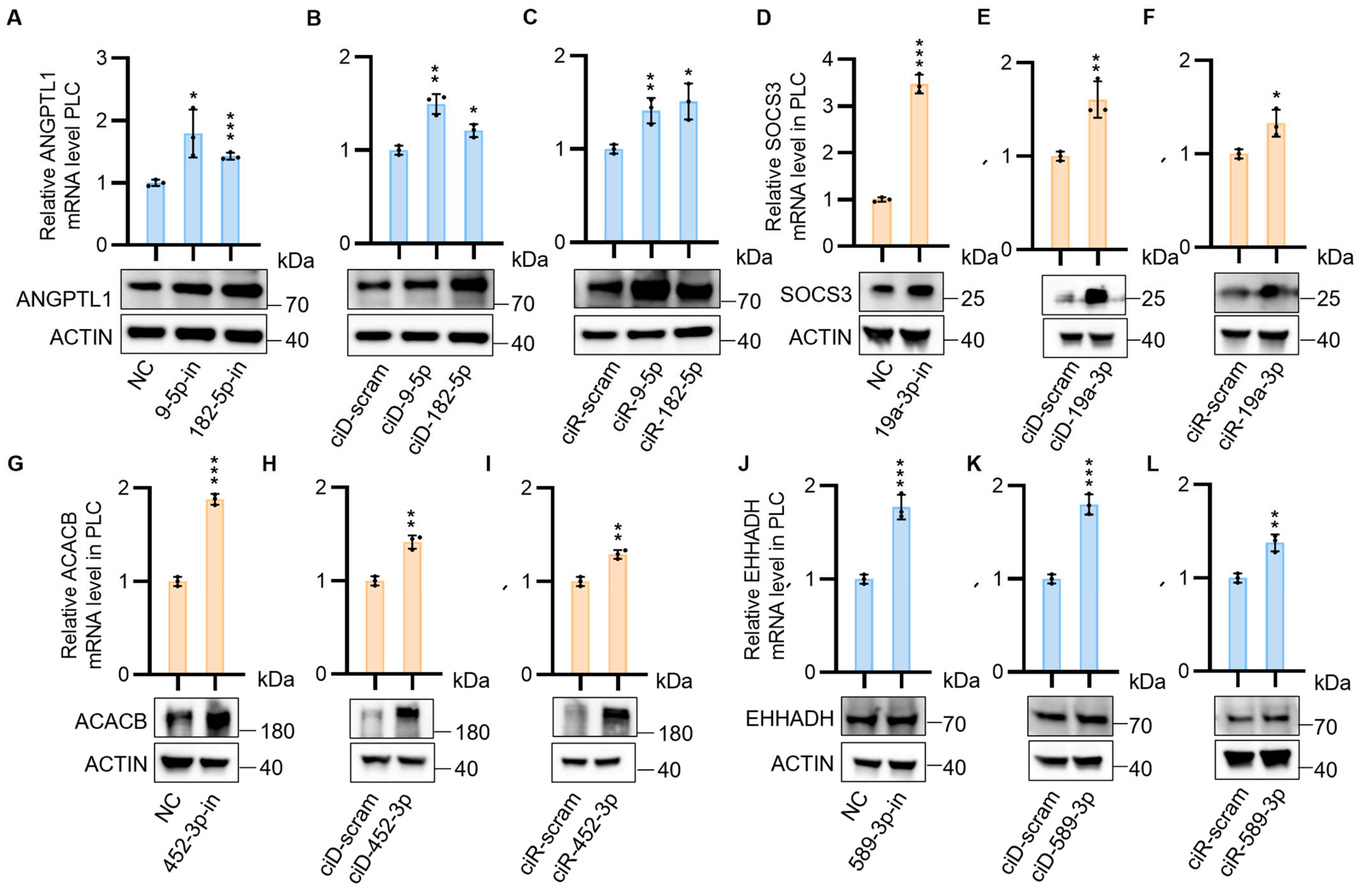

Figure S2. Rescue of ANGPTL1/SOCS3/ACACB/EHHADH Tumor Suppressor Gene Levels in PLC Cells by Inhibitors and circNAs.

(**A**) ANGPTL1 mRNA (RT-qPCR) and protein (Western blot) levels after transfection with miRNA inhibitors (miR-9-5p-in, miR-182-5p-in) targeting oncomiRs in PLC cells. (**B**) ANGPTL1 mRNA (RT-qPCR) and protein (Western blot) levels post-transfection with circDNAs (circDNA-9-5p, circDNA-182-5p) in PLC cells. (**C**) ANGPTL1 mRNA (RT-qPCR) and protein (Western blot) levels following circRNA transfection (circRNA-9-5p, circRNA-182-5p) in PLC cells. (**D**) SOCS3 mRNA (RT-qPCR) and protein (Western

blot) levels after miRNA inhibitor transfection (miR-19a-3p-in) in PLC cells. (**E**) SOCS3 mRNA (RT-qPCR) and protein (Western blot) levels post-circDNA transfection (circDNA-19a-3p) in PLC cells. (**F**) SOCS3 mRNA (RT-qPCR) and protein (Western blot) levels following circRNA transfection (circRNA-19a-3p) in PLC cells. (**G**) RT-qPCR and Western blot analysis of ACACB mRNA and protein levels in PLC cells transfected with miRNA-452-3p-in. (**H**) ACACB mRNA and protein expression in PLC cells transfected with circDNA-452-3p. (**I**) ACACB mRNA and protein expression in PLC cells transfected with circRNA-452-3p. (**J**) RT-qPCR and Western blot analysis of EHHADH mRNA and protein levels in PLC cells transfected with miRNA-589-3p-in. (**K**) EHHADH mRNA and protein expression in PLC cells transfected with circDNA-589-3p. (**L**) EHHADH mRNA and protein expression in PLC cells transfected with circRNA-589-3p. Statistical significance was determined by Student's *t*-tests (n ≥ 3, * $p < 0.05$, ** $p < 0.005$, *** $p < 0.001$).

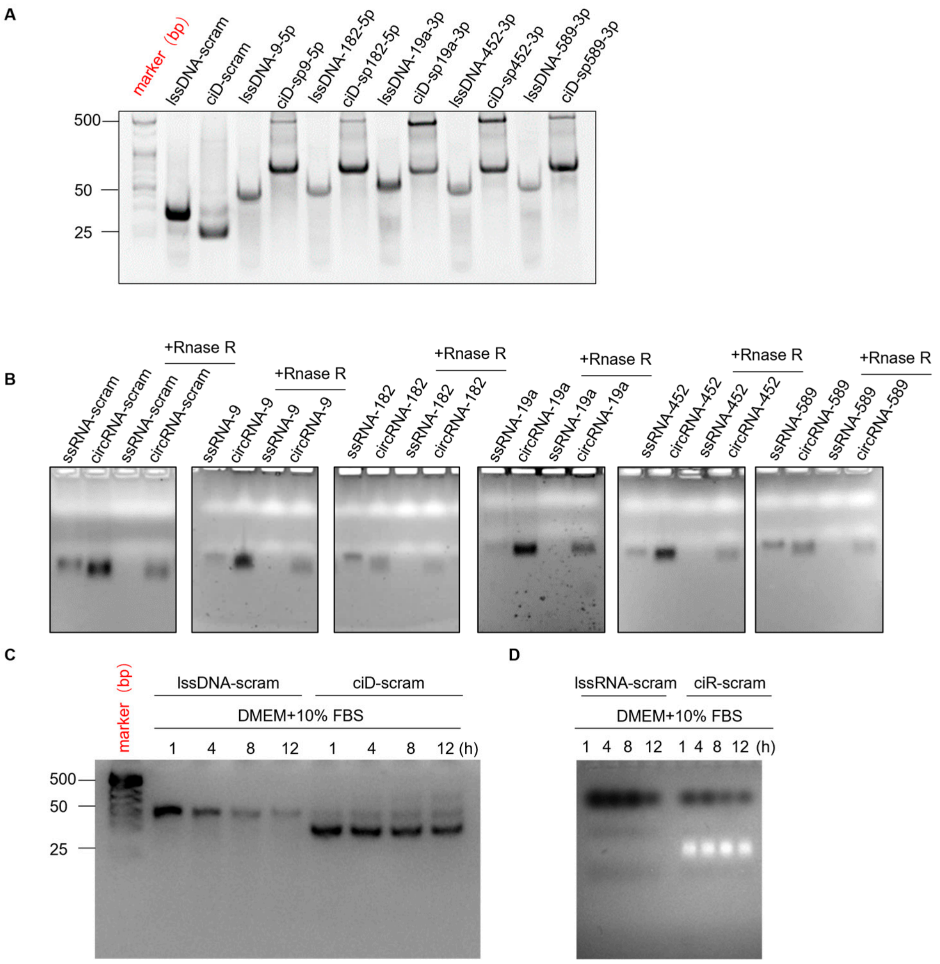

Figure S3. Molecular Characterization and Serum Stability Profiling of CircNAs by Polyacrylamide/Agarose Gel Electrophoresis.

(**A**) Polyacrylamide gel electrophoresis analysis of CircDNAs (CircDNA-9-5p, CircDNA-182-5p, CircDNA-19a-3p, CircDNA-452-3p, CircDNA-589-3p). (**B**) Agarose gel electrophoresis analysis of CircRNAs (CircRNA-9-5p, CircRNA-182-5p, CircRNA-19a-3p, CircRNA-452-3p, CircRNA-589-3p). (**C**) Serum stability assay of lssDNA scram and circDNA scram (40 pmol) incubated in 50 μL DMEM + 10% FBS at 37 °C for 1, 4, 8, and 12 hours, analyzed by 20% polyacrylamide gel electrophoresis. (**D**) Serum stability assay of lssRNA scram and circRNA scram (40 pmol) incubated in 50 μL DMEM + 10% FBS at 37 °C for 1, 4, 8, and 12 hours, analyzed by 2% formaldehyde-denaturing agarose gel electrophoresis.

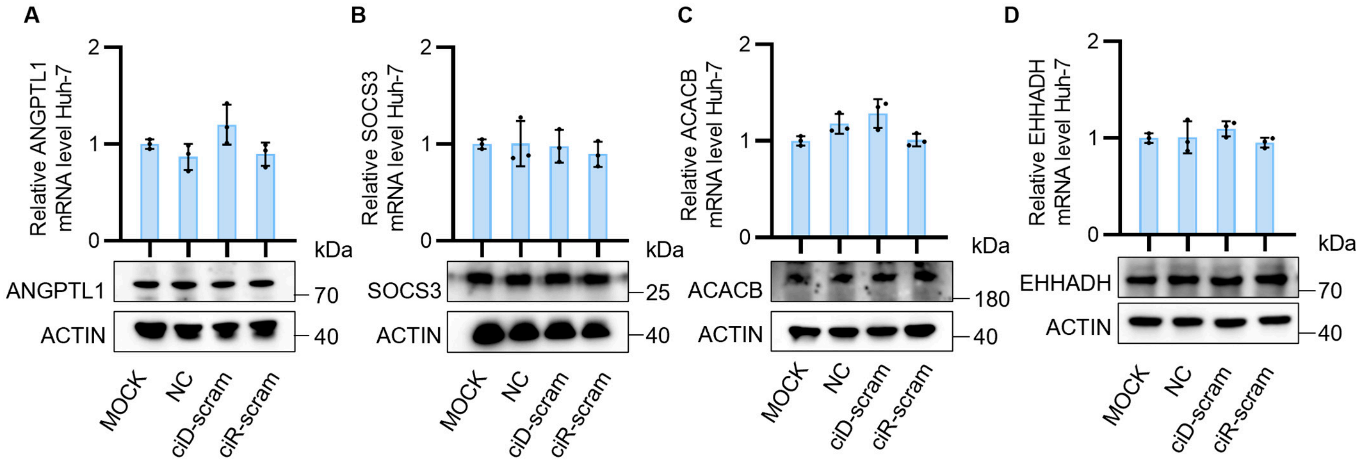

Figure S4. Baseline Characterization of TSGs Expression Across Control Cohorts.

(**A**) RT-qPCR and Western blot analysis of ANGPTL1 mRNA and protein expression in HCC cells following treatment with four control groups: mock, inhibitor-nc, ciD-scram, and ciR-scram. (**B**) RT-qPCR and Western blot analysis of SOCS3 mRNA and protein expression in HCC cells treated with mock, inhibitor-nc, ciD-scram, or ciR-scram. (**C**) mRNA and protein levels of ACACB in HCC cells across control groups (mock, inhibitor-nc, ciD-scram, ciR-scram), assessed by RT-qPCR and Western blot. (**D**) RT-qPCR and Western blot evaluation of EHHADH expression in HCC cells after treatment with mock, inhibitor-nc, ciD-scram, or ciR-scram.

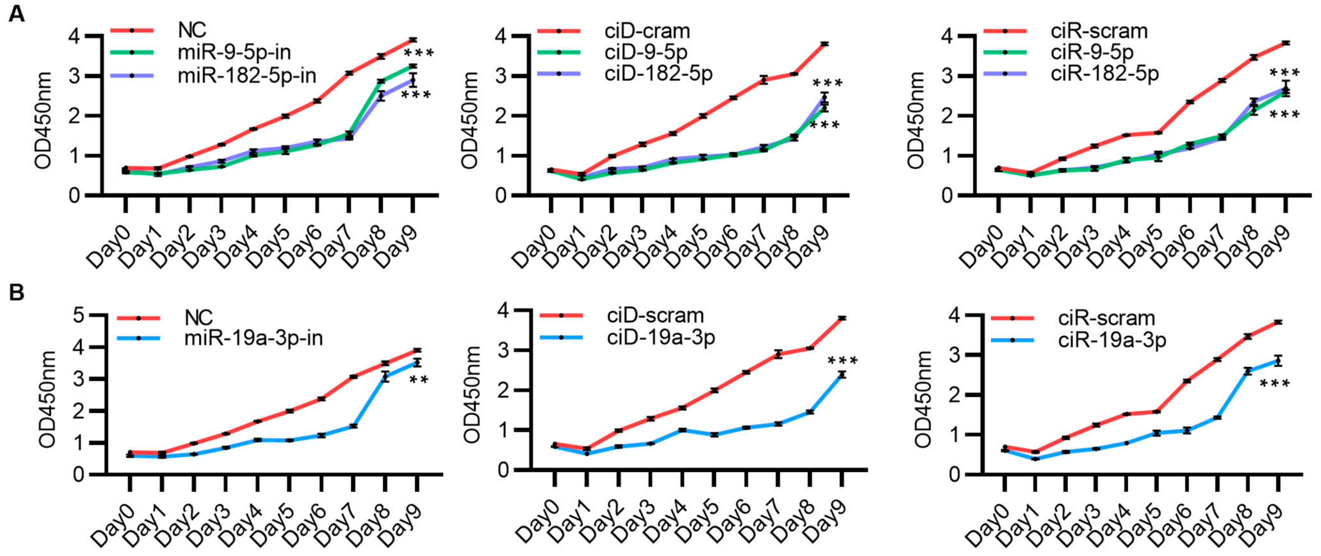

Figure S5. CircNAs Maintains Prolonged Anti-Proliferative Effects in HCC Cells.

(**A,B**) The CCK-8 assay was used to investigate the proliferative effects of miRNA inhibitors, circDNAs, and circRNAs on Huh-7 cell proliferation. Statistical significance was determined by Student's t-tests (n ≥ 3, ***p* < 0.005, ****p* < 0.001).

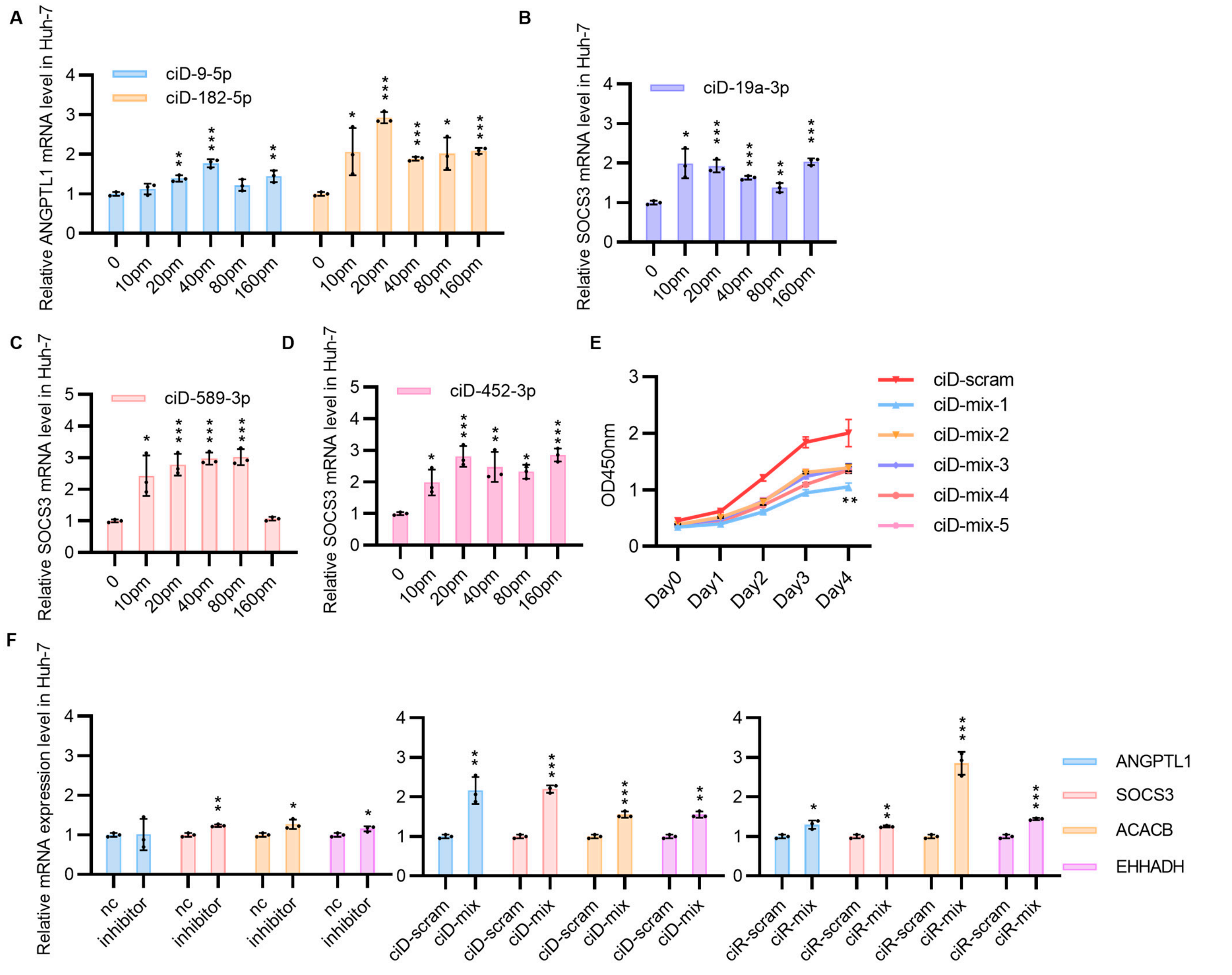

Figure S6. Explore the best ratio and concentration of circDNA mix.

(**A**) Huh-7 cells were treated with escalating concentrations of ciD-9-5p and ciD-182-5p (0 pm, 10 pm, 20 pm, 40 pm, 80 pm, 160 pm), and the mRNA expression levels of ANGPTL1 in HCC cells were quantitated via reverse transcription quantitative polymerase chain reaction (RT-qPCR). (**B**) Huh-7 cells were treated with escalating concentrations of ciD-19a-3p (0 pm, 10 pm, 20 pm, 40 pm, 80 pm, 160 pm), and the mRNA expression levels of SOCS3 in HCC cells were quantitated via reverse transcription quantitative polymerase chain reaction (RT-qPCR). (**C**) Huh-7 cells were treated with escalating concentrations of ciD-452-3p (0 pm, 10 pm, 20 pm, 40 pm, 80 pm, 160 pm), and the mRNA expression levels of ACACB in HCC cells were quantitated via reverse transcription quantitative polymerase chain reaction (RT-qPCR). (**D**) Huh-7 cells were treated with escalating concentrations of ciD-589-3p (0 pm, 10 pm, 20 pm, 40 pm, 80 pm, 160 pm), and the mRNA expression levels of EHHADH in HCC cells were quantitated via reverse transcription quantitative polymerase chain reaction (RT-qPCR). (**E**) The proliferative capacity of HCC cells, following transfection with diverse circDNA mixtures, was evaluated using the Cell Counting Kit-8 (CCK8) assay. (**F**) Huh-7 cells were treated with optimally determined combinations of inhibitor mix, circDNA mix, and circRNA mix. The mRNA expression levels of ANGPTL1, SOCS3, ACACB and EHHADH were quantified using RT-qPCR. Statistical significance was determined by Student’s t-tests (n ≥ 3, * $p < 0.05$, ** $p < 0.005$, *** $p < 0.001$).

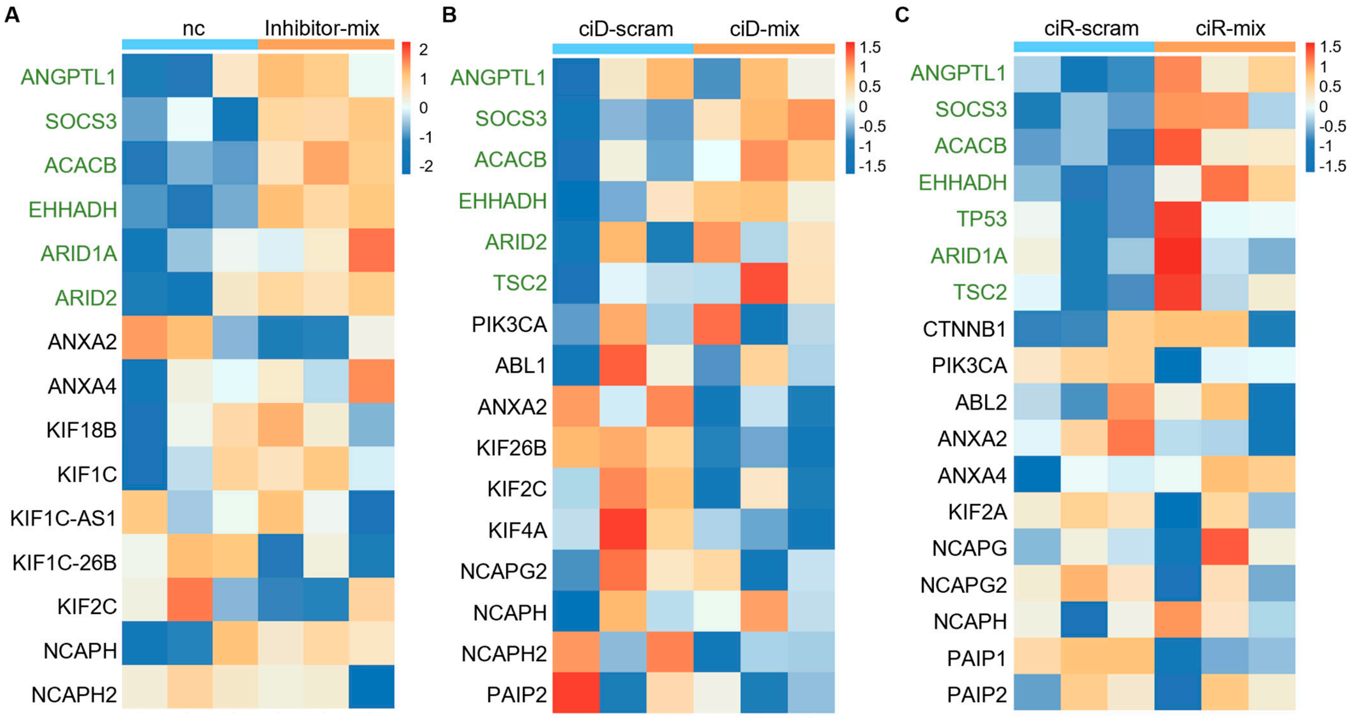

Figure S7. CircNAs Mixes Suppress Classical and Emerging Oncogenes Through Multi-Target Mechanisms.

(**A**) The hierarchical clustering heatmap demonstrates the differential expression of target tumor suppressor genes (TSGs) (*ANGPTL1*, *SOCS3*, *ACACB*, *EHHADH*, *ARID1A*, *ARID2*) and emerging tumor-associated genes (*ANXA2*, *ANXA4*, *KIF18B*, et al.) relative to untreated controls. (**B**) The hierarchical clustering heatmap demonstrates the differential expression of TSGs (*ANGPTL1*, *SOCS3*, *ACACB*, *EHHADH*, *ARID2*, *TSC2*) and emerging tumor-associated genes (*ABL1*, *ANXA2*, *KIF26B*, *KIF2C*, *KIF4A*, et al.) relative to untreated controls. (**C**) The hierarchical clustering heatmap demonstrates the differential expression of TSGs (*ANGPTL1*, *SOCS3*, *ACACB*, *EHHADH*, *TP53*, *ARID1A*, *TSC2*) and emerging tumor-associated genes (*NCAPG2*, *PAIP1*, *ANXA2*, et al.) relative to untreated controls.

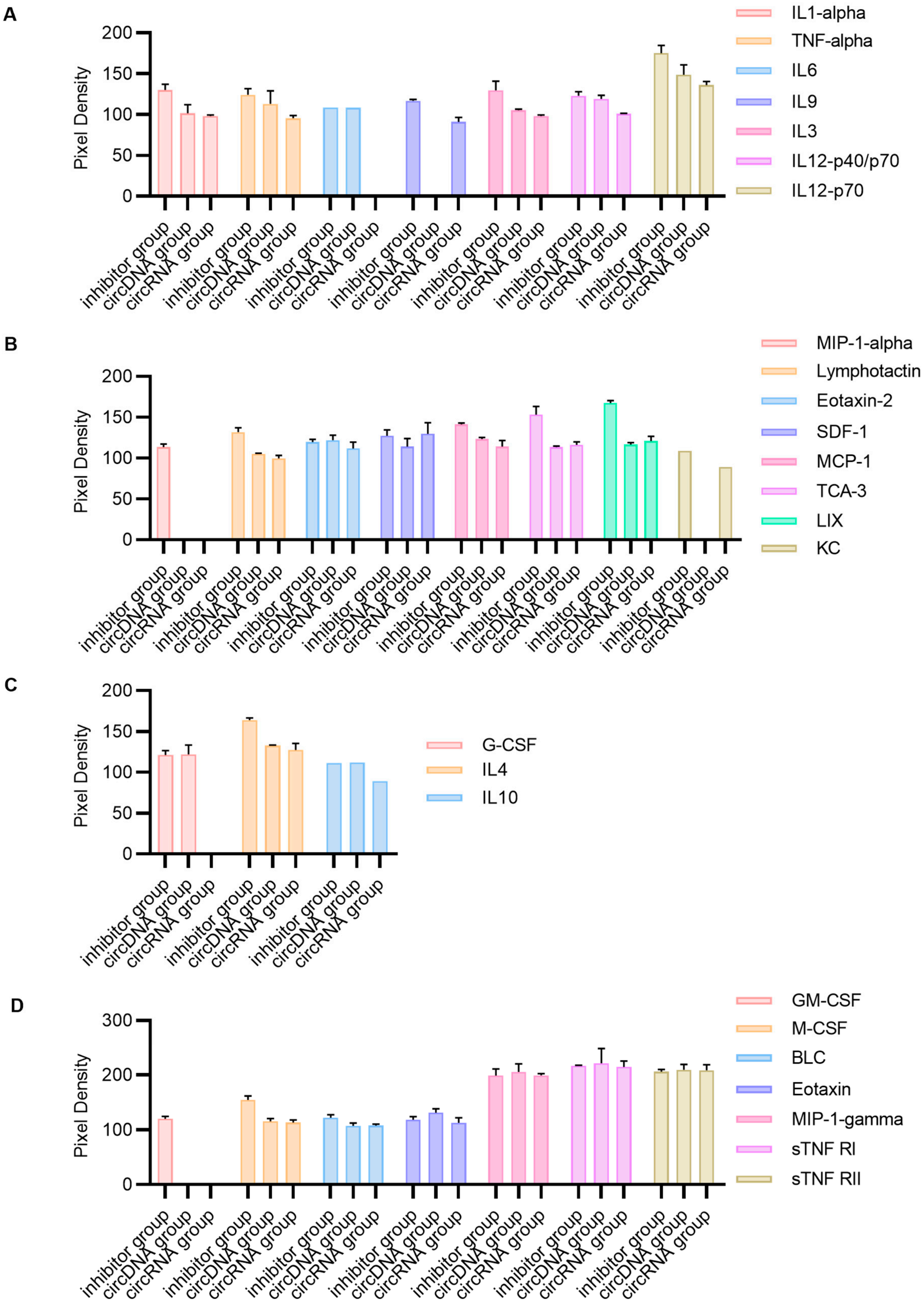

Figure S8. The comparison of the inflammatory activation of three different sponge vectors.

(**A**) Bar Chart Analysis of Pixel Density of Pro-inflammatory Cytokines in inhibitor group, ciD group, and ciR group. (**B**) Bar Chart Analysis of Pixel Density of pro-inflammatory chemokines in inhibitor group, ciD group, and ciR group. (**C**) Bar Chart Analysis of Pixel Density of anti-inflammatory chemokines in inhibitor group, ciD group, and ciR group. (**D**) Bar Chart Analysis of Pixel Density of pro- and anti-inflammatory chemokines in inhibitor group, ciD group, and ciR group.

Supplementary Table S1. Oligomers used in this study.

Name	Application	Sequence
qPCR-ANGPTL1-F	qPCR	AGTGGACACTGGACATTGCAG
qPCR-ANGPTL1-R	qPCR	GCTTCCTCTTTACCATCTGTGG
qPCR-SOCS3-F	qPCR	CATCTCTGTCGGAAGACCGTCA
qPCR-SOCS3-R	qPCR	GCATCGTACTGGTCCAGGAACT
qPCR-ACACB-F	qPCR	CAAGCCGATCACCAAGAGTAAA
qPCR-ACACB-R	qPCR	CCCTGAGTTATCAGAGGCTGG
qPCR-EHHADH-F	qPCR	AAACTCAGACCCGGTTGAAGA
qPCR-EHHADH-R	qPCR	TTGCAGAGTCTACGGGATTCT
Paired primers	circDNA construction	AACAGTACTTTTGTGTAGTACAA
circDNA-scram	circDNA construction	CAAAAGTACTGTTTTTTCATACTTTGTGTAGTACAATTTTTTCATACTT TTGTGTAGTACAATTTTTTTTGTACTACA
circDNA-9-5p	circDNA construction	CAAAAGTACTGTTTTTTTCATCACAGATAACCAAAGATTTCATCAGCT AGATAACCAAAGATTTTTTTGTACTACA
circDNA-182-5p	circDNA construction	CAAAAGTACTGTTTTTTAGTGTGATTCTACATGCCAAATTTTTTATGTG AGTTTACATGCCAAATTTTTTTTGTACTACA
circDNA-19a-3p	circDNA construction	CAAAAGTACTGTTTTTTTCAGTTTGCATGATTGCACATTTTTTTCATTTT GCATGATTGCACATTTTTTTTGtACTACA
circDNA-452-3p	circDNA construction	CAAAAGTACTGTTTTTTCATTACTTCTTTGCAGATGATTTTTTCATTACT TCTTGCAGATGAGTTTTTTTTGTACTACA
circDNA-589-3p	circDNA construction	CAAAAGTACTGTTTTTTTCTGGGAACCGGCATTTGTTCTGATTTTTTTC TGGGAACCGGCATTTGTTCTGATTTTTTTGTACTACA
circ-T7-scram-F	*In vitro* RNA transcription	TAATACGACTCACTATAGGGTTTTTTTCAGTACTTTTGTGTAGTACAAT TTTTTCAGTACTTTTGTGTAGTACAATTTTTTA
circ-T7-scram-R	*In vitro* RNA transcription	TAAAAAATTGTACTACACAAAAGTACTGAAAAAATTGTACTACACA AAAGTACTGAAAAAAACCCTATAGTGAGTCGTATTA
circ-T7-9-F	*In vitro* RNA transcription	TAATACGACTCACTATAGGGTTTTTTTCATACAGCTACCCACCAAAGA TTTTTTTCATACAGCTACCCACCAAAGATTTTTTA
circ-T7-9-R	*In vitro* RNA transcription	TAAAAAATCTTTGGTGGGTAGCTGTATGAAAAAAATCTTTGGTGGGT AGCTGTATGAAAAAAACCCTATAGTGAGTCGTATTA
circ-T7-182-F	*In vitro* RNA transcription	TAATACGACTCACTATAGGGTTTTTTAGTGTGAGTTCTAGCTTGCCAA ATTTTTTAGTGTGAGTTCTAGCTTGCCAAATTTTTTA
circ-T7-182-R	*In vitro* RNA transcription	TAAAAAATTTGGCAAGCTAGAACTCACACTAAAAAATTTGGCAAGCT AGAACTCACACTAAAAAACCCTATAGTGAGTCGTATTA

circ-T7-19a-F	*In vitro* RNA transcription	TAATACGACTCACTATAGGGTTTTTTTCAGTTTTGCACCCTTTGCACATTTTTTTCAGTTTTGCACCCTTTGCACATTTTTTA
circ-T7-19a-R	*In vitro* RNA transcription	TAAAAAATGTGCAAAGGGTGCAAAACTGAAAAAAATGTGCAAAGGGTGCAAAACTGAAAAAAACCCTATAGTGAGTCGTATTA
circ-T7-452-F	*In vitro* RNA transcription	TAATACGACTCACTATAGGGTTTTTTCACTTACTTCACACAGATGAGTTTTTTCACTTACTTCACACAGATGAGTTTTTTA
circ-T7-452-R	*In vitro* RNA transcription	TAAAAAACTCATCTGTGTGAAGTAAGTGAAAAAACTCATCTGTGTGAAGTAAGTGAAAAAACCCTATAGTGAGTCGTATTA
circ-T7-589-F	*In vitro* RNA transcription	TAATACGACTCACTATAGGGTTTTTTTCTGGGAACCGGACCTGTTCTGATTTTTTTCTGGGAACCGGACCTGTTCTGATTTTTTA
circ-T7-589-R	*In vitro* RNA transcription	TAAAAAATCAGAACAGGTCCGGTTCCCAGAAAAAAATCAGAACAGGTCCGGTTCCCAGAAAAAAACCCTATAGTGAGTCGTATTA
